# Sugar and sugar-free liquid formulations of delamanid for patients with rifampicin-resistant TB

**DOI:** 10.5588/ijtld.22.0329

**Published:** 2023-01-01

**Authors:** R. Taneja, M. C. Nahata, J. Scarim, P. G. Pande, A. Scarim, G. Hoddinott, C. L. Fourie, R. K. Jew, H. S. Schaaf, A. J. Garcia-Prats, A. C. Hesseling

**Affiliations:** 1 Global Alliance for TB Drug Development (TB Alliance), New York, NY, USA; 2 Institute of Therapeutic Innovations and Outcomes, Colleges of Pharmacy and Medicine, The Ohio State University, Columbus, OH, USA; 3 JSAS Services Inc, Tucson, AZ, USA; 4 Desmond Tutu TB Centre, Department of Paediatrics and Child Health, Faculty of Medicine and Health Sciences, Stellenbosch University, Tygerberg, South Africa; 5 Metro TB Complex, Department of Health, Pretoria, South Africa; 6 Institute for Safe Medication Practices, Horsham, PA, USA; 7 Department of Pediatrics, University of Wisconsin School of Medicine and Public Health, Madison, WI, USA

**Keywords:** antitubercular, medicine, suspension, DLM

## Abstract

**BACKGROUND:**

Delamanid (DLM) tablets are recommended for the treatment of rifampicin-resistant TB. However, no liquid or dispersible tablet formulation of DLM is currently commercially available for patients with challenges ingesting these tablets. The aim of this study was to develop stable extemporaneous liquid formulations of DLM that can be stored at room temperature for several weeks.

**METHODS:**

DLM tablets were suspended in 1) simple syrup and 2) a specially formulated sugar-free vehicle. These suspensions containing DLM 5 mg/mL were stored in plastic prescription bottles at room temperature or 30°C for 30 days. These suspensions were evaluated for appearance, potency, pH, and microbial counts at Days 0, 15, and 30.

**RESULTS:**

The potency of DLM in each formulation remained at 98–104% of the theoretical concentration for 30 days. The appearance, pH, and microbial count did not change for the sugar-free formulation during the 30-day storage period. Microbial growth, however, was observed in the simple syrup formulation on Day 30 but not on Day 15.

**CONCLUSION:**

DLM can be formulated in sugar or sugar-free suspensions and stored at room temperature or 30°C for at least 15 and 30 days, respectively.

## Abstract (fr)

**CONTEXTE:**

Les comprimés de délamanide (DLM) sont recommandés pour le traitement de la TB résistante à la rifampicine. Cependant, aucune préparation liquide ou sous forme de comprimés dispersibles n’est actuellement disponible pour les patients qui ont du mal à avaler ces comprimés. L’objectif de cette étude était de développer des préparations liquides extemporanées stables de DLM, qui peuvent se conserver à température ambiante pendant plusieurs semaines.

**MÉTHODES:**

Des comprimés de DLM ont été formulés en suspensions dans 1) une préparation de sirop simple et 2) dans une préparation spécifique sans sucre. Ces suspensions de 5 mg/ml de DLM ont été conservées dans des flacons en plastique à température ambiante ou à 30°C pendant 30 jours. L’apparence, le pH, la puissance et le taux de bactéries des suspensions ont été déterminés aux Jours 0, 15 et 30.

**RÉSULTATS:**

La puissance du DLM, dans chaque préparation, est restée à 98–104% de la concentration théorique pendant 30 jours. Pour la préparation sans sucre, l’apparence, le pH et le taux de bactéries n’ont pas varié au cours de la période de conservation de 30 jours. Une croissance bactérienne a, quant à elle, été observée dans les préparations de sirop simple au Jour 30, mais pas au Jour 15.

**CONCLUSION:**

Le DLM peut être préparé en suspensions avec ou sans sucre et conservé à température ambiante ou à 30°C pendant au moins 15 et 30 jours, respectivement.

Rifampicin-resistant TB (RR-TB) continues to be a serious public health threat globally, estimated to cause up to 32,000 incident cases in children <15 years of age each year.^1^ Pediatric evaluation of new and repurposed drugs used for RR-TB treatment has been delayed. Furthermore, lack of age-appropriate formulations continues to be an unmet need for patients unable to swallow tablets or capsules, especially pediatric patients. These challenges result in limited access for children to much needed therapeutic advances.

Delamanid (DLM) is effective and well-tolerated in adult and pediatric patients with RR-TB and is a WHO Group C anti-tuberculosis drug.^2^–^9^ It is available in many countries for oral administration as 50-mg tablets. The WHO has recommended DLM use in children down to age 3 years since 2019, and since 2021 for children of all ages.^9^–^12^ However, no DLM formulation is commercially available for patients who have difficulty swallowing tablets or prefer a liquid formulation instead of tablets, which has limited its uptake in young children. In 2021, a 25-mg dispersible tablet was approved by the European Medicines Authority (EMA) for use in children down to 10 kg of body weight.^13^ Although this dispersible tablet is expected to become more widely available, as of 2021 it was only available through compassionate use; global uptake may take time and it may remain unavailable in some locations. Evidence-based approaches are needed to allow administration of DLM to children using the currently accessible 50-mg tablets until a dispersible tablet is universally available.

This study was designed to develop two stable extemporaneous liquid formulations of DLM to allow dose titration and administration to patients who cannot swallow tablets, ensuring that these extemporaneous formulations can be easily prepared in a pharmacy or dispensary, including in low- or middle-income countries (LMIC) using easily accessible, inexpensive ingredients and minimal equipment, and that these formulations can be stored from room temperature to 30°C for several weeks. Amber plastic prescription bottles were utilized to store the suspensions to prevent photodegradation, which is a potential concern for DLM.

## METHODS

### Preparation of delamanid sugar formulation

Simple syrup was prepared by dissolving 255 g of cane sugar in 135 mL of hot distilled water, followed by equilibrating to room temperature. Ten DLM 50 mg tablets (Deltyba^®^, Otsuka Pharmaceutical, Tokyo, Japan) were ground to a fine powder using a glass mortar and pestle. Twenty mL of simple syrup was added to the mortar and the contents were mixed for 30 sec. An additional 77 mL of simple syrup was added in incremental amounts while mixing thoroughly to bring the volume in the mortar to 100 mL. The contents were mixed well for about 30 sec to obtain a homogeneous suspension of DLM 5 mg/mL. This well-mixed formulation was transferred into an amber 120-mL plastic prescription bottle with a syringe adapter and child-resistant cap (CRC) for storage. The stepwise preparation method is described in Table 1.

**Table 1:** Instructions for the preparation of DLM 5 mg/mL formulation in simple syrup

Instructions for preparing simple syrup (65% w/w) vehicle from cane sugar: Weigh 255 g of food-grade cane sugar into a suitable container Add 135 mL of hot distilled water and mix well until sugar is dissolved Cool syrup to ambient room temperature Instructions for DLM formulation in simple syrup: Grind 10 tablets of DLM (50 mg each) to a fine powder in a glass mortar and pestle Mix powder with a small amount (20 mL added using an oral syringe) of simple syrup vehicle to form a uniform paste Add an additional 77 mL of syrup vehicle in increasing amounts while mixing thoroughly to bring total volume in the mortar to 100 mL Transfer the final contents from the mortar into an appropriately sized amber bottle

DLM = delamanid.

### Preparation of delamanid sugar-free formulation

Thick & Easy^®^ is a widely available modified food starch used as a thickening agent in foods and fluids for patients who experience difficulty swallowing. Thick & Easy powder 11.5 g was mixed with 236 mL of distilled water for 30 sec with a spoon. This mixture was allowed to hydrate for 5 min.

Ten DLM 50 mg tablets, sodium saccharin 125 mg, citric acid 125 mg, potassium sorbate 100 mg, and methylparaben 100 mg were added to a glass mortar, followed by addition of 50 mL of distilled water. The tablets were allowed to disintegrate for 5 min. The disintegrated tablets and powders were mixed with a pestle to form a uniform mixture. Finally, 47 mL of Thick & Easy vehicle was added to bring the volume in the mortar to 100 mL. The contents were mixed well to obtain a homogeneous suspension of DLM 5 mg/mL with no lumps present. The suspension was transferred into an amber 120-mL plastic prescription bottle with a syringe adapter and CRC. The stepwise preparation method is described in Table 2. A general technique for preparing the suspension and withdrawing doses is shown in the Figure.

**Table 2:** Instructions for the preparation of DLM 5 mg/ml formulation in a sugar-free vehicle

Instructions for preparing Thick & Easy® sugar-free vehicle: Measure 236 mL (8 oz) of distilled water into a suitable container Add 11.5 g of Thick & Easy® powder Mix well with a spoon for 30 seconds and let the mixture sit for at least 5 min before use Mix again just prior to use Instructionsfor preparing DLM sugar-free formulation: Add 10 tablets of DLM (50 mg each) to a glass mortar and pestle Add 100 mg of methyl paraben, 100 mg of potassium sorbate, 125 mg of citric acid and 125 mg of sodium saccharin to the tablets in the mortar Add 50 mL of distilled water into the mortar using an oral syringe and let the mixture sit for 5 min Mix the completely disintegrated tablets using a glass pestle to form a uniform mixture Using an oral syringe, add 47 mL of Thick & Easy® sugar-free vehicle to bring total volume in the mortar to 100 mL and mix to form a uniform suspension Transfer the final contents from the mortar into an appropriately sized amber bottle

DLM = delamanid.

**Figure:**
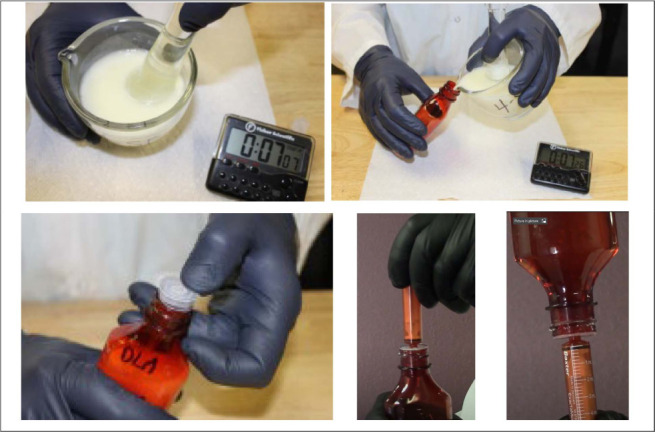
Preparation of delamanid suspensions.

### Storage stability of formulations

Twelve bottles of each DLM formulation were prepared as described above. Six bottles were stored at room temperature and the remaining six bottles at 30°C for 30 days. For each formulation, three bottles were designated for potency testing, one for appearance and pH testing, one for microbial testing, and one was extra as a backup, if needed. Aliquots were withdrawn from each bottle at the designated time-point for testing.

### Appearance and pH

A 25-mL sample of each well-mixed suspension formulation was transferred to a 30-mL beaker on Days 0, 15, and 30 and examined by visual inspection and under a light microscope at 40X and 100X magnifications to document any changes in appearance. The pH of the two formulations was measured on Days 0, 15, and 30 using a digital pH meter calibrated with pH 4 and 7 buffers.

### Measurement of delamanid potency

A stability-indicating high-performance liquid chromatographic (HPLC) method was developed and verified by conducting forced degradation studies on DLM, including exposure to light, acid, base and oxidation. This method was used to determine potency of DLM in both formulations on Days 0, 15, and 30. Three aliquots were withdrawn from each of the three bottles for a total of *n* = 9 at each timepoint.

The diluent was prepared with 1:1 water and acetonitrile and equilibrated at room temperature. A 0.25 mg/mL DLM stock standard solution was prepared by dissolving DLM powder in methanol and diluting with the diluent to 0.025 mg/mL for the working standard solution. The sample solution was prepared by diluting 5 mL of the DLM suspension with methanol to a concentration of 0.25 mg/mL and further diluting with the diluent to 0.025 mg/mL. A 10-mL aliquot of this mixture was filtered through a 0.45 μm polypropylene membrane syringe filter. The filtrate was analyzed using HPLC.

The mobile phase consisted of HPLC grade water, acetonitrile and trifluoroacetic acid (650:350:1). A C18 HPLC column 4.6 mm × 100 mm, 3.5 μm was used at 40°C. The mobile phase flow rate was 1.5 mL/min and ultraviolet detection was at 223 nm.

Ten μL of working standard solution was injected into the chromatographic system. The area under the curve (AUC) for the DLM peak eluting at approximately 8 min was recorded as a percentage of theoretical concentration. Ten μL of the diluent was injected as a blank to ensure no interference with the DLM peak. A relative standard deviation of <2% was documented with five consecutive injections of the working standard to show the system reproducibility for potency. During the run, working standard solution was injected to demonstrate HPLC system stability. The DLM peak AUC agreed with the system suitability average within 2%. The stability of the working standard and the filter suitability were documented. The HPLC method was linear from 0.0125 to 0.0375 mg/mL of DLM. It was specific and stability-indicating that the peaks resulting from the forced degradation of DLM did not interfere with the DLM peak.

### Evaluation of microbial growth

Suitability studies were conducted to evaluate and verify that the methods described in the United States Pharmacopeia (USP)<60>,USP<61>,andUSP<62> were suitable to recover the specific microorganisms in the two DLM formulations. The verified procedures were used to enumerate and determine the absence of microorganisms in the two DLM suspensions at Days 0, 15, and 30. The specific microorganisms tested in the formulations were *Staphylococcus aureus* (American Type Culture Collection [ATCC] 6538), *Pseudomonas aeruginosa* (ATCC9027), *Candida albicans* (ATCC10231), *Escherichia coli* (ATCC8739), *Aspergillus brasiliensis* (ATCC16404), *Burkholderia cepacia* (ATCC25416), and *Zygosaccharomyces rouxii* (ATCC28253) which included those tested for extemporaneous vehicles.^14^ OnemL ofsamplefrom each DLM suspension bottle was diluted with 9 mL of neutralizing broth. For positive and negative controls, 10 mL of neutralizing broth was utilized. The inoculum of each pathogen was prepared using phosphate buffer and serial dilution made with Tryptic Soy broth. A volume of 0.1 mL of the inoculum at a selected 10^3^ colony-forming unit (CFU)/mL concentration was spiked into the sample, positive control, and negative control tubes separately and mixed well. One mL of the spiked sample, positive control, and negative control were placed on agar plates (bacteria on Tryptic Soy Agar [TSA] plates, andyeast andmoldonSabouraud Dextrose Agar [SDA] plates). The TSA plates were incubated at 30–35°C for 3–5 days and SDA plates at 20–25°C for 5–7 days. The colonies for each microorganism were counted.

Following pre-enrichment, appropriate plates were streaked and incubated for specific microorganisms per USP<62> at the specified temperatures for specified times. After incubation, the presence or absence of the target organism was verified against positive controls.

### Outcome measures

The criteria for acceptable liquid formulations of DLM included ease of preparation of the formulations using widely accessible and affordable ingredients in LMIC with a high prevalence of TB. Appropriate storage conditions were defined as the storage of suspensions in readily available plastic prescription bottles at room temperature and 30°C. The formulations were considered to be homogeneous and potency was acceptable if the measured DLM concentrations were within 10% of the theoretical value.^15^ A smooth, uniform, light yellow suspension with no lumps or clumps with visual inspection and no change in visual or microscopic appearance during the study period were considered acceptable formulations. Conformance with the USP<1111> limits for total aerobic microbial counts (TAMC) and total yeast and mold counts (TYMC), and absence of specified microorganisms in the formulations were considered acceptable (Supplementary Data).

### Ethics statement

Ethics statement is not applicable to this study, as this was not a human subjects study.

## RESULTS

Both DLM 5 mg/mL suspensions prepared in sugar and sugar-free vehicles were uniform, smooth, and could be easily redispersed with gentle shaking. The visual and microscopic appearance of the two formulations were off white to light yellow in color. This appearance did not change during the 30-day study period at room temperature or 30°C.

As shown in Table 3, the DLM potency ranged from 97.9% to 101.1% and from 98.9% to 104.2% of the theoretical concentration in the sugar and sugar-free formulations, respectively, demonstrating that the aliquots withdrawn were homogeneous. The pH ranged from 5.08 to 6.02 in the sugar formulation and from 4.17 to 4.35 in the sugar-free formulation.

**Table 3:** Potency of delamanid in two liquid formulations

Study day	Theoretical concentration (*n* = 9)			

	Sugar formulation		Sugar-free formulation	
	
	Room temperature % mean ±SD	30°C % mean ±SD	Room temperature % mean ±SD	30°C % mean ± SD
0	99.19 ± 0.38	99.46 ± 0.69	100.34 ± 0.59	100.56 ± 0.79
15	99.17 ± 0.49	99.42 ± 0.55	100.39 ± 0.91	101.16 ± 0.98
30	99.90 ± 0.64	100.13 ± 0.50	102.18 ± 0.61	102.79 ± 0.78

SD = standard deviation.

Microbial tests for six specific microorganisms and *Burkholderia cepacia* complex showed no growth during the 30-day study period in the two formulations at room temperature and at 30°C. TAMC met the USP<1111> limits for aqueous oral liquids in the sugar and sugar-free formulations (Table 4). TYMC met the USP<1111> limits for aqueous oral liquids in the sugar-free formulation. On Day 30, however, the TYMC exceeded the USP limits in the simple syrup formulation stored at room temperature and at 30°C. Therefore, this formulation was acceptable for storage up to Day 15 but not Day 30.

**Table 4:** Results of microbial limits tests of two delamanid liquid formulations *

Microbial counts	Microbial limits test results					

	Sugar formulation (RT and 30°C)			Sugar-free formulation (RT and 30°C)		
	
	Day 0 cfu/g	Day 15 cfu/g	Day 30 cfu/g	Day 0 cfu/g	Day 15 cfu/g	Day 30 cfu/g
TAMC (RT)	30	<10	155	<10	<10	<10
TAMC (30°C)	20	10	80	<10	<10	<10
TYMC (RT)	<10	<10	150^†^	<10	<10	<10
TYMC (30°C)	<10	<10	320^†^	<10	<10	<10

* Per USP<1111>, acceptable criteria for microbiological quality of oral aqueous liquids are TAMC <200 cfu/g and TYMC <20 cfu/g, and absence of *E. coli*.

† Sugar formulation failed microbial quality criteria for TYMC on Day 30 at RT and 30°C.

RT=room temperature; TAMC=total aerobic microbial count; TYMC=total yeast and mold count; USP=United States Pharmacopeia.

## DISCUSSION

DLM has been approved in many countries as a 50-mg tablet, and more recently, by the EMA as a 25-mg dispersible tablet formulation, for administration two times a day for 24 weeks.^13^ The 25-mg dispersible tablets are to be dispersed in water using 10–15 mL per tablet and ingested immediately. Thereafter, an additional 10–15 mL of water per dispersible tablet must be added to the cup to ensure that the remaining suspension is dispersed, and then ingested. This is a high volume for small children, although smaller volumes are likely adequate and are being used in the field. Lack of potable water would further complicate the dose preparation with dispersible tablets.

We performed this study to develop a feasible DLM dose administration alternative for children and older patients with dysphagia, primarily for settings in which the dispersible tablets are not available. These formulations are ready to administer and avoid the need of dispersion for dose preparation two times a day for 24 weeks.

Two extemporaneous oral liquid formulations of DLM (sugar and sugar-free) were developed. The sugar-free liquid formulation would serve the needs of patients with sugar intake restrictions. These formulations can be used effectively for dose titration, especially young children, and for patients with dysphagia. No dose preparation is required prior to administration. We investigated the stability of the formulations at two different temperatures to accommodate the different storage temperatures expected in the field. We limited the stability studies to 30 days to meet the beyond-use date requirements for USP<795>.^16^ The assigned beyond-use date was confirmed by actual stability testing of microbial count and absence of specific organisms.

The two liquid preparations of DLM for oral administration met the requirements of desirable compounded formulations. Susceptibility to oxidation and heat has been cited in literature as potential challenges with breaking DLM tablets.^17^ Our results showed that 5 mg/mL DLM was stable in simple syrup and sugar-free liquid formulations after preparation and storage at both ambient room temperature and 30°C. The microbial count did not change for the sugar-free formulation during storage. However, the simple syrup formulation met the microbial limits at 15 days but did show growth at Day 30. The use of potassium sorbate and methylparaben as preservatives in the sugar-free liquid formulation ensured no substantial growth of microorganisms during the storage period of 30 days. Citric acid was added to the sugar-free formulation to maintain the pH below 6 which is optimum for antimicrobial activity of potassium sorbate.^18^ Growth of microorganisms in the simple syrup formulation on Day 30 can be explained by lack of preservatives in the formulation. The sugar-free liquid formulation provides a choice for patients with diabetes or on calories restriction. The low volume required (5 mL for a 25-mg dose) is a useful feature of these extemporaneous formulations.

We intentionally utilized easily accessible and affordable ingredients (cane sugar, Thick & Easy) to prepare the formulations to maximize their use in countries with a high prevalence of TB. Cane sugar can be substituted with powdered sugar. Alternatively, commercially available simple syrup can be used. The DLM suspensions were pharmaceutically elegant and easily resuspended with gentle shaking. A few tablet coating fragments were observed in the simple syrup preparation, but it did not impact the homogeneity of the suspension. No substantial changes occurred in the appearance, pH, or potency of these formulations upon storage for the duration of the study. The plastic bottles used for storing the suspensions were representative of the prescription dispensing bottles routinely used in pharmacies, including in LMIC.

## CONCLUSIONS

DLM formulations as sugar and sugar-free suspensions were easily prepared with ingredients that are widely available and affordable in LMIC. DLM was chemically and physically stable during the 30-day study period at room temperature and 30°C. USP limits were met for microbial counts during the storage for 15 days in the sugar formulation and 30 days in the sugar-free formulation. Thus, liquid formulations of DLM can be extemporaneously prepared and stored for 15 days in simple syrup and 30 days in the sugar-free formulation. Either of the two liquid formulations may be used to treat RR-TB in adult and pediatric patients who have difficulty swallowing tablets and have the potential to substantially improve access to DLM for children in support of the WHO’s new recommendation for its use in children of all ages.
